# Anesthesia Management of a Patient With Patent Ductus Arteriosus: A Case Report of Eisenmenger Syndrome

**DOI:** 10.7759/cureus.115890

**Published:** 2026-09-07

**Authors:** Sophia Kontak, Jose L Diz Ferre, Anthony L Zaki, Edward G Soltesz, Sabry S Ayad

**Affiliations:** 1 Outcomes Research Consortium, Cleveland Clinic, Cleveland, USA; 2 Department of Thoracic and Cardiovascular Surgery, Cleveland Clinic, Cleveland, USA; 3 Heart, Vascular, and Thoracic Institute, Cleveland Clinic, Cleveland, USA; 4 Department of Anesthesiology, Cleveland Clinic Fairview Hospital, Cleveland, USA

**Keywords:** eisenmenger syndrome, hemodynamic monitoring, laparoscopic technique, patent ductus arteriosus, pulmonary hypertension

## Abstract

Patent ductus arteriosus is a congenital heart defect characterized by the persistence of a fetal connection between the pulmonary artery and the aorta, resulting in abnormal blood flow depending on its size. When left uncorrected, it can lead to pulmonary arterial hypertension and Eisenmenger syndrome. This disorder is characterized by the following triad: large intra- or extracardiac congenital defect with an initial systemic-to-pulmonary shunt, pulmonary arterial hypertension with shunt reversal (right-to-left) or bidirectional shunting, and hypoxemia with cyanosis. Understanding this challenging pathophysiology is important when delivering anesthesia, especially during intraoperative acute anesthesia events. We report on successful anesthesia management of a 30-year-old female patient with Eisenmenger syndrome who underwent an elective bilateral laparoscopic salpingectomy. This case report highlights the anesthesia considerations to avoid exacerbation of the shunt.

## Introduction

A cardiac shunt is a congenital or added abnormal blood flow inside or outside the heart. Multiple causes of shunt include atrial septal defects, ventricular septal defects, and patent ductus arteriosus (PDA) [1]. By the eighth week of fetal life, the proximal branch pulmonary arteries arise from the proximal portions of the sixth pair of aortic arches, while the distal portion of the left sixth arch persists as the ductus arteriosus [2]. Normally, it remains patent, connecting the pulmonary artery and the aorta until it disappears after birth, but failing to disappear results in abnormal blood flow depending on its size [2]. The chronic presence of a shunt (left-to-right) causes high pulmonary blood flow that induces pulmonary vasculature remodeling. This leads to increased pulmonary vascular resistance and pulmonary arterial hypertension (PAH). When this process is perpetuated, the magnitude of these hemodynamic changes becomes eventually the Eisenmenger syndrome, which is characterized by shunt reversal (right-to-left) [3]. In this syndrome, reversal of the shunt and entry of deoxygenated blood into the systemic circulation result in hypoxemia. We describe the anesthesia considerations to prevent increasing shunt in a patient who underwent a bilateral laparoscopic salpingectomy with this complex pathophysiology. This article was previously posted to the Authorea preprint server on September 1, 2024 [4].

## Case presentation

We report a case of a 30-year-old Hispanic patient, height 155 cm and weight 55 kg, who presented for an elective bilateral laparoscopic salpingectomy after request for sterilization. Her significant medical history included bicuspid aortic valve, PAH, and polycythemia due to PDA, and Eisenmenger syndrome. Her outpatient medication included spironolactone 25 mg QD, treprostinil 3.25 mg TID [5], macitentan 10 mg QHS, and sildenafil 80 mg daily. Additionally, she was on long-term controller medication with fluticasone propionate/salmeterol for asthma and rizatriptan to palliate migraine headaches.

Despite experiencing side effects such as facial flushing and occasional hypotension from PAH treatment, the patient has been tolerating therapy well. Since previous visits with pulmonology, the patient has remained clinically stable with no reported hospitalizations or emergency room visits for decompensated heart failure or exacerbation of asthma. She remained free of lower extremity edema, exertional palpitations or near-syncope, and no longer had episodes of peripheral cyanosis with significant exertion. During pre-anesthesia testing, she reported good exercise tolerance up to 5.5 metabolic equivalent of task. Regarding her secondary polycythemia, she has been consulting hematology and deferring phlebotomy for hyperviscosity due to a lack of symptoms.

Previous echocardiogram showed a 65% ejection fraction. The right ventricle was dilated with moderate hypertrophy of the right ventricular (RV) free wall (Figures 1, 2) and normal systolic function. Abnormal motion of the interventricular septum consistent with high RV pressure and volume overload was noted. Due to insufficient tricuspid regurgitation signal, the estimated RV systolic pressure (RVSP) was not reported, but a prior RVSP was 40 mmHg. A bicuspid aortic valve with no aortic regurgitation was present. There was trace mitral, tricuspid, and pulmonary regurgitation with a peak gradient of 11 mmHg. Estimated right atrial pressure was 8 mmHg based on inferior vena cava assessment. No changes were noted in the previously documented bi-directional shunt between the aorta and pulmonary artery (Figure 3). Previous Swan-Ganz catheter assessment revealed a mean pulmonary artery pressure of 99 mmHg and Wood Units pulmonary vascular resistance of 12.15. The electrocardiogram also showed right atrial enlargement and RV hypertrophy.

**Figure 1:**
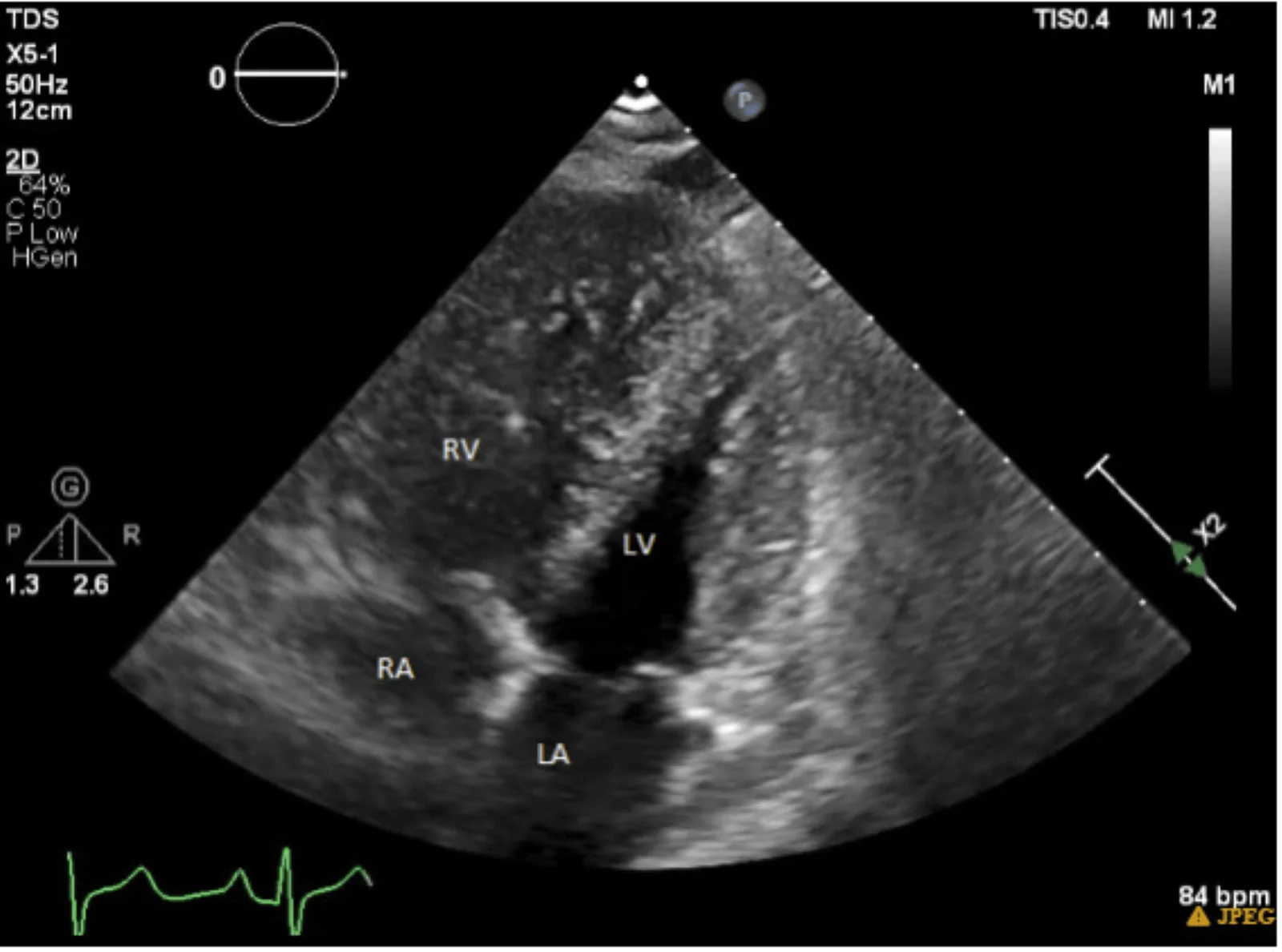
Echocardiogram four-chamber view showing hypertrophic and dilated right ventricle. LA, left atrium; LV, left ventricle; RA, right atrium; RV, right ventricle.

**Figure 2:**
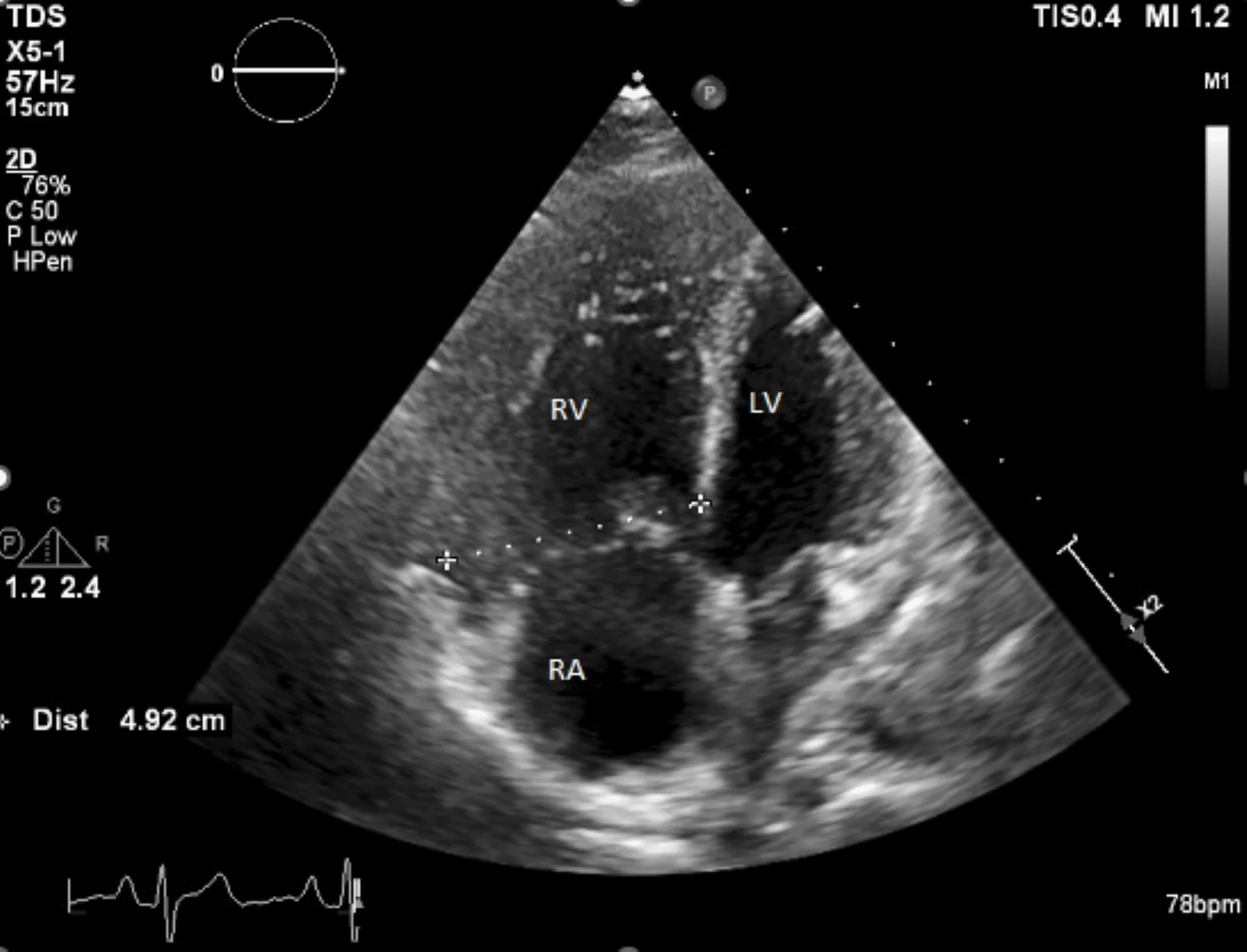
Echocardiogram four-chamber view showing right atrium dilation and 4.92 cm dilation of the hypertrophied right ventricle. LV, left ventricle; RA, right atrium; RV, right ventricle.

**Figure 3:**
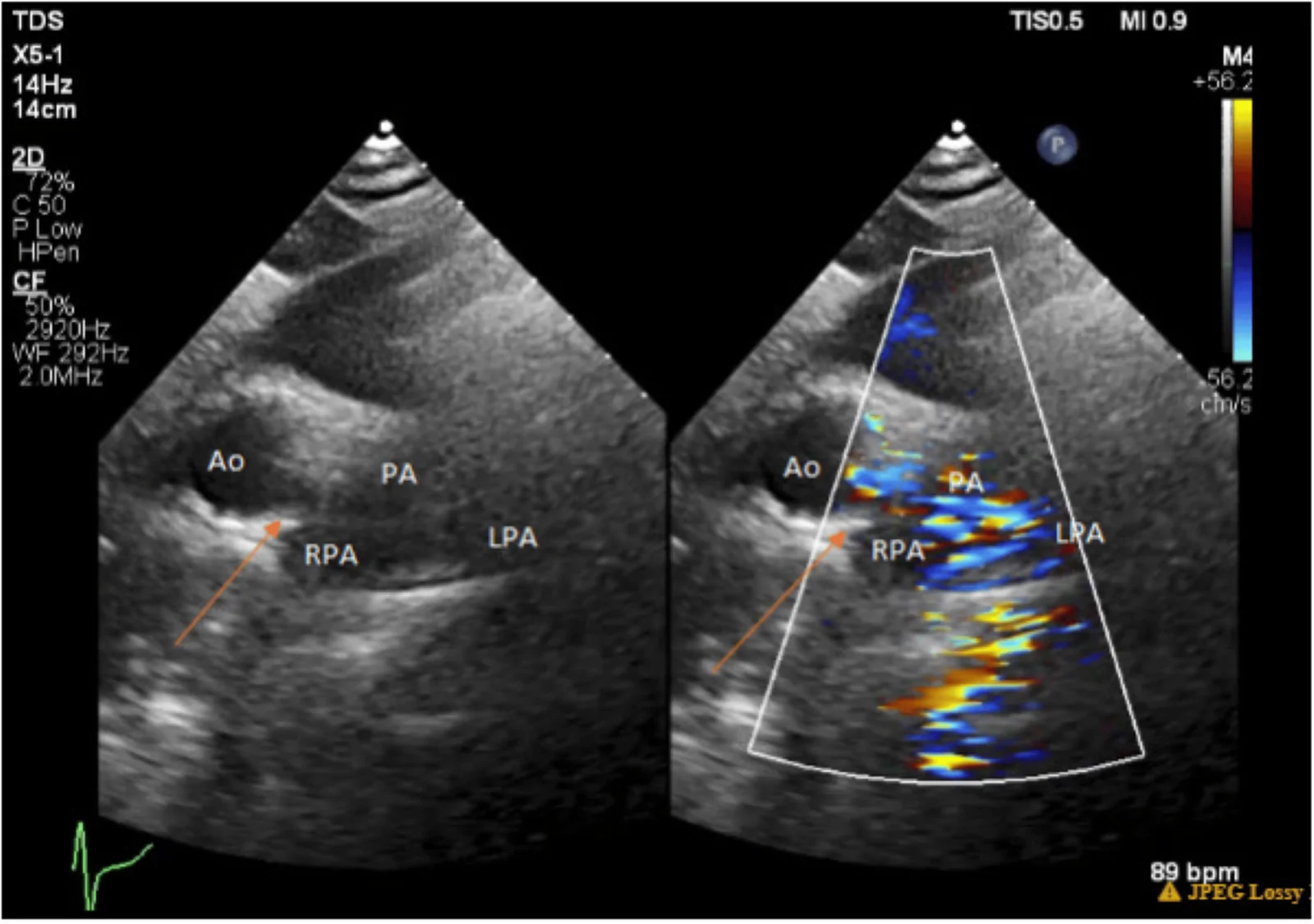
Parasternal pulmonary artery bifurcation view with orange arrow showing uncorrected patent ductus arteriosus. Ao, aorta; LPA, left pulmonary artery; PA, pulmonary artery; RPA, right pulmonary artery.

Except for a typical PDA murmur, physical examination was found with normal findings. The preoperative comprehensive metabolic panel showed an increase in potassium levels with no apparent clinical correlation (Table 1). Preoperative vital signs were blood pressure 118/87 mmHg, pulse 91, respiratory rate 18, and SpO_2_ 94% on room air. Anesthesia considerations included ultrasound-guided arterial line for continuous systolic, diastolic, mean arterial pressure (MAP) and wave morphology monitoring, as well as arterial blood gases (ABGs) as needed. This was placed in the right radial artery before induction in the operating room. Electrocardiogram, bispectral index, blood pressure cuff, saturation, and temperature were also adopted. Before intubation, ABGs were obtained, displaying the results in Table 2. General anesthesia was performed with endotracheal intubation using etomidate 16 mg, rocuronium 30 mg, lidocaine 60 mg, and fentanyl 50 mcg.

**Table 1:** Comprehensive metabolic panel Preoperative comprehensive metabolic panel results

Component		Result	Reference range and unit
Protein		6.3	6.3-8.0 g/dL
Albumin		3.4 (low)	3.9-4.9 g/dL
Calcium		8.9	8.5-10.2 mg/dL
Bilirubin		0.4	0.2-1.3 mg/dL
Alkaline phosphatase		107	34-123 U/L
Aspartate aminotransferase		32	13-35 U/L
	Comment: Results may be falsely increased due to interference from hemolysis. Suggest reorder as clinically indicated.		
Alanine aminotransferase		13	7-38 U/L
Glucose		131 (high)	74-99 mg/dL
	Comment: The American Diabetes Association (ADA) provides guidance for cutoff values for fasting glucose and random glucose. The ADA defines fasting as no caloric intake for at least eight hours. Fasting plasma glucose levels between 100 and 125 mg/dL indicate an increased risk of diabetes (prediabetes). Fasting plasma glucose levels greater than or equal to 126 mg/dL meet the criteria for the diagnosis of diabetes. In the absence of unequivocal hyperglycemia, results should be confirmed by repeat testing. In a patient with classic symptoms of hyperglycemia or hyperglycemic crisis, a random plasma glucose level greater than or equal to 200 mg/dL meets the criteria for the diagnosis of diabetes [6].		
Blood urea nitrogen		21	7-21 mg/dL
Creatinine		1.00 (high)	0.58-0.96 mg/dL
Sodium		138	136-144 mmol/L
Potassium		5.4 (high)	3.7-5.1 mmol/L
Chloride		105	97-105 mmol/L
Carbon dioxide		14 (low)	22-30 mmol/L
Anion gap		19 (high)	9-18 mmol/L
Estimated glomerular filtration rate		78	≥60 mL/min/1.73 m²
	Comment: Estimated glomerular filtration rate (eGFR) is calculated using the 2021 CKD-EPI creatinine equation. This equation uses serum creatinine, sex, and age as parameters. The creatinine assay has traceable calibration to isotope dilution-mass spectrometry. Refer to KDIGO guidelines for clinical interpretation. In patients with unstable renal function, e.g., those with acute kidney injury, the eGFR may not accurately reflect actual GFR.		

**Table 2:** Arterial blood gases Arterial blood gases before intubation

Component	Result	Reference range and unit
Potential of hydrogen (PH)	7.423	Arterial 7.350-7.450 units
Partial pressure of carbon dioxide (CO_2_)	33.5 (abnormal)	Arterial 35.0-45.0 mmHg
Partial pressure of oxygen (O_2_)	69 (abnormal)	Arterial 80-105 mmHg
Base excess point-of-care testing (POCT)	-3 (abnormal)	-2 to 3 mmol/L
Bicarbonate (POCT)	21.9 (abnormal)	22.0-26.0 mmol/L
Total CO_2_ (POCT)	23	23-27 mmol/L
Saturation O_2_ (POCT)	94 (abnormal)	95%-98%
Sodium (POCT)	138	138-146 mmol/L
Potassium (POCT)	5.9 (abnormal)	3.5-4.9 mmol/L
Glucose (POCT)	112 (abnormal)	74-99 mg/dL
Ionized Ca (POCT)	1.24	1.12-1.32 mmol/L
Hemoglobin (POCT)	<>	12.0-17.0 g/dL
Hematocrit (POCT)	>75 (abnormal)	38%-51%

However, after intubation, the pulse increased to 130 bpm and MAP decreased to 66 mmHg. The ECG tracing showed sinus rhythm. Phenylephrine 300 mcg intravenous bolus and ephedrine 5 mg were administered, as well as an infusion of phenylephrine 0.01% at 0.27 mL/kg/h along with lactated Ringer (LR) solution at 1.36 mL/kg/h for plasma volume expansion. The MAP was maintained above 70 mmHg. After the incision, insufflation pressure was set to 10 mmHg with minimal Trendelenburg, and no further blood pressure variation during laparoscopy or with positional changes was noted. A total of 600 mL LR solution, ephedrine 20 mg, phenylephrine 1350 mcg, and sodium bicarbonate 50 mEq were given. Ventilation parameters remained stable at tidal volume of 350 mL, respiratory rate of 18 breaths/min, SpO_2_ of 94% on 93% FiO_2_, positive inspiratory pressure of 20 cm H_2_O, positive end-expiratory pressure of 4 cm H_2_O, and end-tidal CO_2_ of 30 mmHg. The urine output was 300 mL, and the estimated blood loss was 5 mL.

Vital signs also remained stable within normal limits during the maintenance with sevoflurane 2.3%. Before the patient was extubated, albuterol 6 puffs were administered to mitigate bronchospasm and sugammadex 200 mg as a muscle relaxant reversal. The procedure was successfully completed without any major anesthesia events, and the patient was discharged in stable condition on the same day. She was encouraged to follow up on her severe pulmonary hypertension with her pulmonologist.

## Discussion

Anesthesia was successfully administered and adapted to the Eisenmenger syndrome. Despite the risk of radial thrombosis, advanced hemodynamic monitoring was crucial to effectively prevent problematic systemic hypotension in this patient with PDA, PAH, and Eisenmenger syndrome. This condition posed a challenging scenario where complex pathophysiology could trigger unexpected complications. For this reason, anesthesia care was based on close monitoring of vital signs and early signs of shunt increase, and attention to maintain optimal respiratory and hemodynamic status was essential.

Eisenmenger syndrome is a complex and rare complication of different congenital heart diseases. Hemodynamic monitoring is critical due to a high risk of complications in these patients [7,8]. The main goal was to avoid acidosis and vasodilation to ensure a relatively high systemic pressure that reduces right-to-left shunt caused by pulmonary hypertension [9]. Invasive MAP monitoring and cardiovascular stable agents such as etomidate were used during induction. Phenylephrine infusion, ephedrine bolus, and expansion with LR solution were used to preserve the blood pressure within the normal range. In this case, phenylephrine was the drug of choice due to its selective alpha-adrenergic effect with minimal cardiac inotropy or chronotropy compared with other vasopressors such as norepinephrine. This also helped minimize the use of ephedrine to maintain a normal pulse and was particularly important in a context where a high heart rate could worsen the shunt and cause hypoxemia.

Since the PH was normal with low bicarbonate, low base excess, high anion gap, and hyperkalemia, the ABG was interpreted as a mixed disorder with primary metabolic acidosis and also primary respiratory alkalosis, considering that the PaCO₂ (33.5) is less than Winter's Expected PaCO₂ (42.4) = (1.5 × 22.9) + 8 (± 2). Sodium bicarbonate was also used to address the acidosis component. This component raised concerns about the risks of peripheral vasodilation leading to desaturation in the context of shunt due to uncorrected PDA and Eisenmenger syndrome hemodynamics. Despite spironolactone as an alternative explanation for the ABG findings and the lack of evidence for bicarbonate in patients with intraoperative metabolic acidosis [10], the priority was to avoid increased shunt via the vasodilatory effects of metabolic acidosis such as low cardiac contractility and more hyperkalemia [11].

## Conclusions

In summary, pharmacological strategies must be tailored to the patient's physiopathology. Advanced hemodynamic monitoring may be considered even for low-risk surgeries to avoid sudden complications caused by Eisenmenger syndrome. In this altered physiology, prevention of systemic hypotension and acid-base disruptions is key to avoid further shunt.
